# Reward insensitivity is associated with dopaminergic deficit in rapid eye movement sleep behaviour disorder

**DOI:** 10.1093/brain/awac430

**Published:** 2022-11-17

**Authors:** Thomas R Barber, Kinan Muhammed, Daniel Drew, Kevin M Bradley, Daniel R McGowan, Johannes C Klein, Sanjay G Manohar, Michele T M Hu, Masud Husain

**Affiliations:** Nuffield Department of Clinical Neurosciences, University of Oxford, Level 6, West Wing, John Radcliffe Hospital, Oxford, OX3 9DU, UK; Nuffield Department of Clinical Neurosciences, University of Oxford, Level 6, West Wing, John Radcliffe Hospital, Oxford, OX3 9DU, UK; Department of Experimental Psychology, University of Oxford, Anna Watts Building, Radcliffe Observatory Quarter, Oxford, OX2 6GG, UK; Nuffield Department of Clinical Neurosciences, University of Oxford, Level 6, West Wing, John Radcliffe Hospital, Oxford, OX3 9DU, UK; Department of Experimental Psychology, University of Oxford, Anna Watts Building, Radcliffe Observatory Quarter, Oxford, OX2 6GG, UK; Wales Research and Diagnostic PET Imaging Centre, Cardiff University, School of Medicine, University Hospital Wales, Cardiff CF14 4XN, UK; Department of Medical Physics and Clinical Engineering, Oxford University Hospitals NHS Trust, Churchill Hospital, Oxford, OX3 7LE, UK; Department of Oncology, University of Oxford, Oxford OX3 7DQ, UK; Nuffield Department of Clinical Neurosciences, University of Oxford, Level 6, West Wing, John Radcliffe Hospital, Oxford, OX3 9DU, UK; Nuffield Department of Clinical Neurosciences, University of Oxford, Level 6, West Wing, John Radcliffe Hospital, Oxford, OX3 9DU, UK; Department of Experimental Psychology, University of Oxford, Anna Watts Building, Radcliffe Observatory Quarter, Oxford, OX2 6GG, UK; Nuffield Department of Clinical Neurosciences, University of Oxford, Level 6, West Wing, John Radcliffe Hospital, Oxford, OX3 9DU, UK; Nuffield Department of Clinical Neurosciences, University of Oxford, Level 6, West Wing, John Radcliffe Hospital, Oxford, OX3 9DU, UK

**Keywords:** reward, dopamine, Parkinson’s disease, REM sleep behaviour disorder

## Abstract

Idiopathic rapid eye movement sleep behaviour disorder (iRBD) has now been established as an important marker of the prodromal stage of Parkinson’s disease and related synucleinopathies. However, although dopamine transporter single photon emission computed tomography (SPECT) has been used to demonstrate the presence of nigro-striatal deficit in iRBD, quantifiable correlates of this are currently lacking. Sensitivity to rewarding stimuli is reduced in some people with Parkinson’s disease, potentially contributing to aspects of the neuropsychiatric phenotype in these individuals. Furthermore, a role for dopaminergic degeneration is suggested by the fact that reward insensitivity can be improved by dopaminergic medications. Patients with iRBD present a unique opportunity to study the relationship between reward sensitivity and early dopaminergic deficit in the unmedicated state.

Here, we investigate whether a non-invasive, objective measure of reward sensitivity might be a marker of dopaminergic status in prodromal Parkinson’s disease by comparing with SPECT/CT measurement of dopaminergic loss in the basal ganglia. Striatal dopaminergic deficits in iRBD are associated with progression to Parkinsonian disorders. Therefore, identification of a clinically measurable correlate of this degenerative process might provide a basis for the development of novel risk stratification tools.

Using a recently developed incentivized eye-tracking task, we quantified reward sensitivity in a cohort of 41 patients with iRBD and compared this with data from 40 patients with Parkinson’s disease and 41 healthy controls. Patients with iRBD also underwent neuroimaging with dopamine transporter SPECT/CT. Overall, reward sensitivity, indexed by pupillary response to monetary incentives, was reduced in iRBD cases compared with controls and was not significantly different to that in patients with Parkinson’s disease. However, in iRBD patients with normal dopamine transporter SPECT/CT imaging, reward sensitivity was not significantly different from healthy controls. Across all iRBD cases, a positive association was observed between reward sensitivity and dopaminergic SPECT/CT signal in the putamen. These findings demonstrate a direct relationship between dopaminergic deficit and reward sensitivity in patients with iRBD and suggest that measurement of pupillary responses could be of value in models of risk stratification and disease progression in these individuals.

## Teaser

REM sleep behaviour disorder is an important marker of the prodromal phase of Parkinsonian disorders. Barber *et al.* demonstrate that RBD patients with established dopaminergic deficits have blunted pupillary responses to reward. Pupillary reward sensitivity may therefore be a marker of prodromal dopaminergic decline.

## Introduction

Blunting of pupillary responses to reward has been demonstrated in Parkinson’s patients using oculomotor tasks that measure pupillary changes to reward anticipation,^1,2^ a phenomenon considered to play an important role in the neuropsychiatric phenotype of these patients.^1–5^ Pupillary reward sensitivity (pRS) can be increased by pharmacological stimulation of dopaminergic pathways, in keeping with the established role of dopaminergic transmission in reward evaluation.^1^ While these findings suggest that reward insensitivity might be a marker of dopaminergic deficit in Parkinsonian disorders, direct evidence for this is lacking.

A population in whom the link between reward sensitivity and dopamine depletion is of particular interest is patients with idiopathic rapid eye movement sleep behaviour disorder (iRBD). This parasomnia is a highly specific marker of the prodromal phase of degenerative synucleinopathies, including idiopathic Parkinson’s disease, dementia with Lewy bodies (DLB) and, less frequently, multiple system atrophy. Idiopathic RBD often develops many years before the onset of motor disease or dementia, and substantial degeneration of striatal dopaminergic neurons occurs during this prodromal period.^6–9^ Patients with iRBD therefore present an unparalleled opportunity to assess the relationship between reward sensitivity and dopaminergic deficit in the early stages of disease, and without the potentially confounding effects of dopaminergic medications used in most patients with manifest Parkinson’s.

When measured in iRBD patients, neuroimaging evidence of a dopaminergic deficit is one of the strongest predictors of near-term conversion to a clinically overt synucleinopathy.^7,10^ However, despite the frequent coexistence of a wide range of motor and non-motor symptoms in these individuals,^11^ none have been shown to relate to underlying dopaminergic deficit.^12^ Being able to estimate striatal dopamine integrity with a clinical test could therefore have important implications for risk stratification in iRBD patients.

This study investigated the extent to which reward sensitivity is impaired in iRBD patients and how this relates to underlying dopaminergic degeneration. Objective quantification of reward sensitivity was achieved using a previously characterized oculomotor task, in which pupillary responses to monetary cues are measured.^1^ This technique is based on the observation that pupils dilate to forthcoming rewarding stimuli and that the magnitude of the physiological response increases with the level of the potential reward on offer.^13^ Studies in healthy control subjects, as well as those with established Parkinson’s disease and small vessel disease, have shown this to be a useful method of quantifying reward sensitivity, which is also independent of autonomic dysfunction and motor preparation.^1,2,14^ pRS in iRBD patients was compared with that in Parkinson’s patients and healthy controls. Alongside this, we quantified clinical apathy and depression to explore their relationship with reward sensitivity.

Dopaminergic deficit was assessed in iRBD patients using ioflupane single-photon emission computed tomography with CT-based attenuation correction (DaT SPECT/CT), which labels presynaptic dopamine transporters and thus quantifies the integrity of striatal dopaminergic synapses.^15^ We hypothesized that iRBD patients with evidence of dopaminergic deficit on imaging would show greater pupillary reward insensitivity than those with normal dopaminergic imaging.

## Materials and methods

### Participants

The study was approved by the local ethics committee, written consent was obtained from all subjects, and the protocol followed the principles of the Declaration of Helsinki. In total, 41 patients with iRBD were recruited prospectively from the Discovery cohort of the Oxford Parkinson’s Disease Centre.^16^ iRBD was confirmed by polysomnography and subjects were excluded if a secondary cause for RBD was present. All iRBD patients underwent pupil reward testing and DaT SPECT/CT brain imaging. The mean interval between imaging and ocular testing was 22 days. Ocular metrics from 40 patients with Parkinson’s disease and 41 healthy age matched controls were collected separately using the same protocol and have been previously published.^1^ Patients with Parkinson’s disease were recruited from clinics in the Oxfordshire area. Control participants were recruited from a volunteer database and were also screened to exclude neurological or psychiatric conditions. Control and Parkinson’s subjects did not undergo polysomnography. All Parkinson’s patients were either drug naïve or tested in the ‘off’ state, with L-dopa having been withdrawn overnight (except for the data presented in Fig. 4D, where Parkinson’s patients were tested separately in both ‘off’ and ‘on’ medication states). Participants were screened for visual problems that might affect task performance, and corrective glasses or contact lenses were worn if required.

To assess the relationship of neuropsychiatric features with pupil responses and dopaminergic deficit, respectively, we measured apathy using the Lille Apathy Rating Scale (LARS),^17^ and depression using the Beck Depression Inventory.^18^ Motor Parkinsonism was assessed by a neurologist experienced in movement disorders using part III of the Unified Parkinson’s Disease Rating Scale (MDS-UPDRS).^19^

### Oculomotor pupillary task

**Figure 1:**
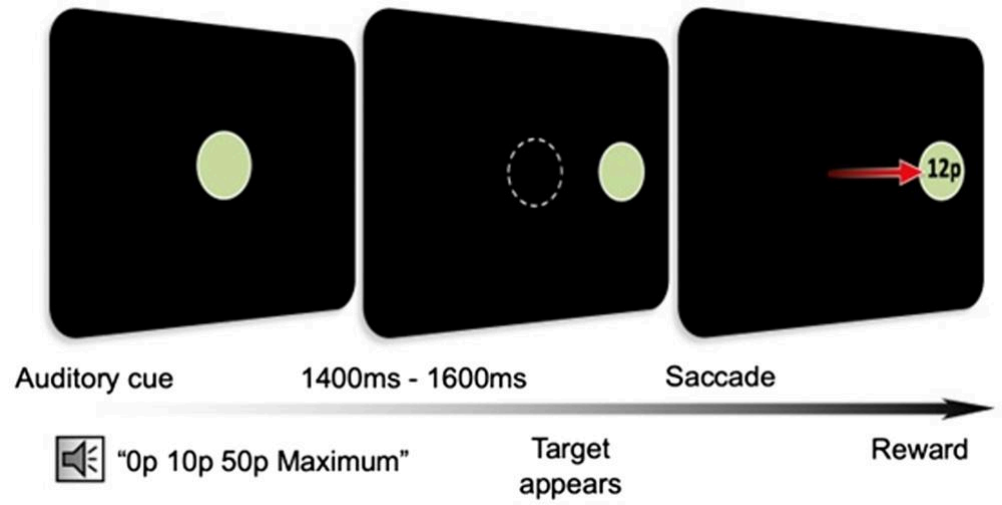
**Oculomotor paradigm schematic representation of the eye tracking task (adapted from Muhammed *et al.*).** ^1^ Participants heard an auditory cue that informed them of the maximum reward available for each trial: ‘0p, 10p or 50p maximum’. After a variable delay of 1400, 1500 or 1600 ms, the central fixation disc disappeared and a new target disc appeared. Participants were rewarded according to reaction time, with the reward obtained displayed within the target disc in pence.

The eye-tracking task was an extensively tested paradigm devised by Muhammed *et al.* (Fig. 1).^1^ An infra-red eye tracker was used to measure changes in pupil diameter in response to an on-screen task. The task involved repeated trials where the participant was required to make a saccadic eye movement in response to a visual target. For each trial, the participant had the possibility of receiving a monetary reward up to a specified maximum value. The actual reward received was a percentage of the maximum offered, calculated according to reaction time such that faster performance resulted in a higher percentage of the maximum reward obtained. Each trial commenced with fixation on a disc in the centre of the screen, at which point baseline pupil size was measured. After 500 ms, a recorded voice is heard informing the participant of the maximum reward available for that trial, either 0p, 10p or 50p maximum (p denotes pence, in pound sterling currency). After a randomly variable delay of 1400 to 1600 ms, the central disc is replaced by a new target that appears randomly either to the left or right of centre at 11 degrees eccentricity, to which the subject must redirect their gaze. The achieved reward is displayed on the screen at the end of each trial in pence. The objective for participants was to obtain as much monetary reward as possible. Pupillary dilation on anticipation of reward during the fore-period was measured. Participants performed 270 trials in five blocks of 54, with 90 trials in total at each of the three reward levels. Each block lasted 3 min, meaning the testing took ∼15 min to complete. All participants that took part in the study had complete recordings successfully captured.

#### Calculation of eye-tracking metrics

Processing of the saccadic and pupillary data is described fully in Muhammed *et al.*^1^ Pupil dilatation was assessed in three ways. First, baseline pupil size was calculated to assess autonomic tone at the pupillary muscles so that background pupil responsiveness could be compared across groups. This was recorded in EyeLink units (EyeLink 1000, SR Research). Second, pupillary dilatation (proportional change from baseline) was calculated at each reward level. The metric pRS was calculated as the average proportional change of pupil size from baseline in response to the 50p reward minus the 0p reward during a set 1000 ms epoch. The time period of interest for measurement of pupillary response was 1400–2400 ms after the auditory reward cue, which was selected based on previous literature to allow enough time for the effects of each reward on the pupil to separate.^1–3,13,14^ This metric assessed the extent to which anticipated rewards modulated pupil dilatation. Pupillary arousal to stimuli was also assessed: this was calculated by averaging pupil proportional change across all three reward levels during the 1400–2400 ms time epoch of interest and this metric was used to evaluate the average pupil arousal response of participants to stimuli irrespective of value. Oculomotor variables were assessed to ensure consistent task performance between subgroups of iRBD patients. Reaction time (RT) was calculated as the time from target onset to completion of a saccade. Saccadic peak velocity was determined as the maximum velocity during a saccade to target.

### Neuroimaging

DaT SPECT/CT scans were acquired using a standard clinical protocol at the Department of Nuclear Medicine, Churchill Hospital, Oxford. Potassium iodide 120 mg was administered 1 h before and 24 h after injection of ^123^I-ioflupane to block thyroid uptake. Subjects were injected with 185 MBq ±10% of ^123^I-ioflupane (provided as DaTSCAN injection, GE Healthcare). SPECT/CT images were acquired on a Discovery 670 hybrid gamma camera (GE Healthcare, Haifa) 3 h post-injection. SPECT acquisition parameters: 120 projections, 30 s per projection, 128 × 128 matrix. CT parameters: 16 slices, helical acquisition, 120 kV, 40 mA and noise index 30. SPECT data were reconstructed using HERMES Hybrid Recon (HERMES Medical Solutions) OSEM, 15 iterations, four subsets with attenuation correction from CT, collimator resolution recovery and Monte Carlo scatter correction.

**Figure 2:**
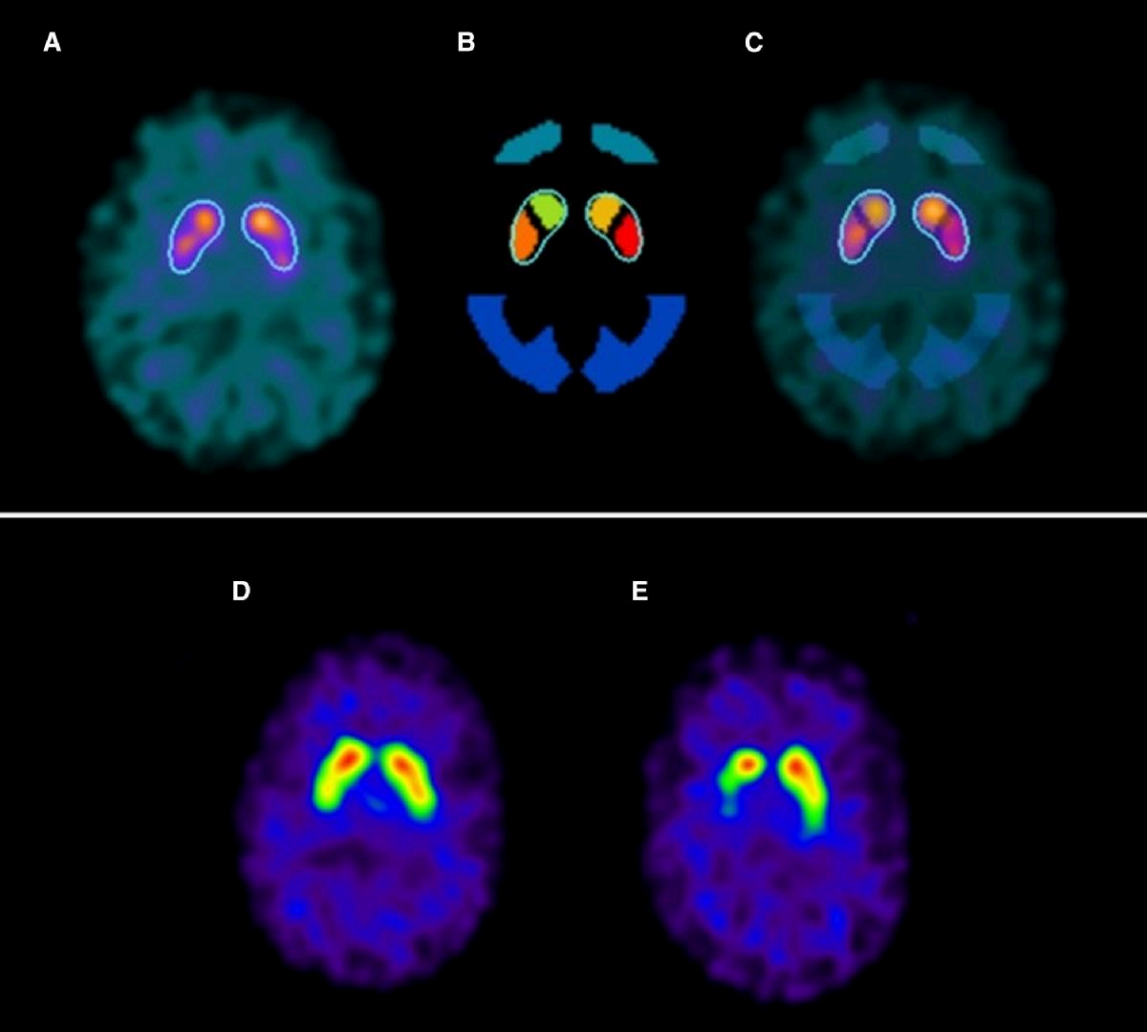
**DaT SPECT/CT imaging in RBD patients.** (**A**) Example striatal ROI registered to DaT SPECT/CT. (**B**) ROI used in the calculation of striatal SURs. Regions used were putamen [posterior ROIs within the striatal outline, shown in red (left putamen) and orange (right putamen)] and occipital reference (dark blue). (**C**) ROI superimposed on an example DaT SPECT/CT image. (**D** and **E**) Example DaT SPECT/CT images from two RBD patients with normal (**D**) and abnormal (**E**) imaging. The abnormal image (**E**) demonstrates asymmetric signal loss in the putamen, typical of Parkinson’s disease.

SPECT/CT imaging data were analysed using BRASS software (HERMES Medical Solutions). Reconstructed images for each patient were registered to a standard template including regions of interest (ROI) for the caudate and putamen on each side (Fig. 2A–C). Uptake ratios were calculated for these ROI using a standard occipital reference region. Dichotomization of RBD patients into those with normal or abnormal imaging was based on the descriptive reports by a Consultant Nuclear Medicine Radiologist, who was blinded to all clinical data other than age and sex (Fig. 2D and E). This assessment considers the pattern of distribution of the SPECT/CT signal and the expected signal for the participant’s age, as well as the absolute signal values. Only scans assessed to be definitely abnormal were classed as abnormal; those with borderline findings were included in the normal category.

### Statistical methods

Differences in baseline clinical characteristics between groups were assessed using pairwise comparisons with independent samples *t*-tests. Variation in pupil responses was assessed using a repeated-measures ANOVA with the three different reward levels (0p, 10p and 50p) as the within-subjects factor, and group categories as between-subjects factors. A linear regression was also performed using the pupillary metrics described above as the dependent variable, and either group category or DaT SPECT/CT putamen signal as independent predictor variables. Baseline pupil size, age and gender were used as covariates in between-groups analyses to control for any effect of these variables. Linear mixed-effects models were used to look at the effect of pRS and pupillary arousal on mean putamen DaT SPECT/CT signal. Significance was taken as *P*-values of <0.05. Statistics were calculated using MATLAB and SPSS v.27.

### Data availability

Access to the data that support the findings of this study may be requested by application to the Oxford Parkinson’s Disease Centre Data Access Committee. Initial enquiries can be made to the corresponding author.

## Results

### Baseline characteristics

The baseline characteristics of the participants are shown in Table 1. There were no significant group-wise differences in age between Parkinson’s disease, iRBD and control groups. The iRBD group had a higher proportion of males than control or Parkinson’s groups, in keeping with the known male predominance seen in iRBD cohorts.^7^ There were no significant differences in age (*P* = 0.60), MDS-UPDRS III score (*P* = 0.80), Beck Depression Inventory score (*P* = 0.54) or LARS (*P* = 0.94) between iRBD patients with normal versus abnormal DaT SPECT imaging. In line with previous studies,^11^ iRBD and Parkinson’s patients were significantly more apathetic than control participants (LARS score: iRBD patients versus controls, *P* < 0.001; Parkinson’s disease patients versus controls, *P* < 0.001), and significantly more depressed than control subjects (Beck Depression Inventory score: iRBD versus controls, *P* < 0.001; Parkinson’s versus controls, *P* < 0.001). There was no difference in the degree of apathy (*P* = 0.48) or depression (*P* = 0.30) between iRBD and Parkinson’s disease patients.

**Table 1:** Baseline demographics and clinical scores of the included participants

	Controls	All RBD patients	iRBD normal DaT	iRBD abnormal DaT	Parkinson’s disease patients
*n*	41	41	23	18	40
Male/female	25/16	40/1	22/1	18/0	26/15
Age, years	64.8 (10.25)	65.2 (7.71)	65.7 (8.87)	64.4 (6.09)	66.4 (5.91)
LARS apathy score	−28.8 (4.06)	−21.3 (5.89)	−21.2 (5.23)	−21.3 (6.76)	−22.4 (8.13)
Beck Depression Inventory score	4.8 (5.27)	11.3 (9.38)	12.4 (10.38)	9.9 (8.00)	13.1 (7.19)
MDS-UPDRS, part III	n/a	5.4 (3.58)	5.6 (3.30)	5.3 (4.00)	20.62 (9.77)

Numbers in brackets represent standard deviation. MDS-UPDRS III = Unified Parkinson’s Disease Rating Scale, Part 3 (motor examination).

### Pupillary responses

**Figure 3:**
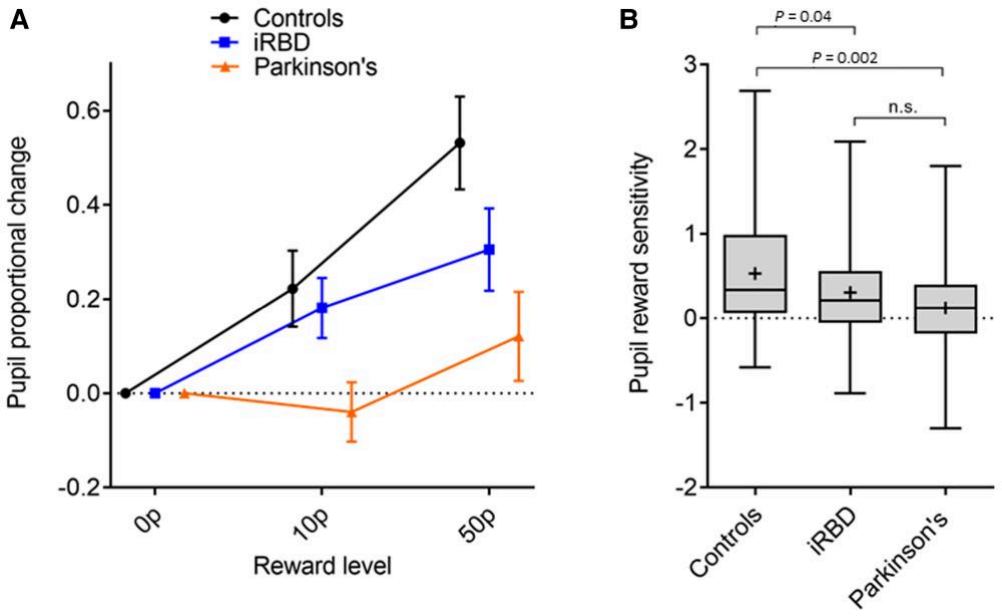
**Pupillary response to reward in controls, RBD and Parkinson’s disease patients.** (**A**) Proportional pupil changes at each reward level across healthy controls, RBD and Parkinson’s disease patients, with each group normalized to the 0p baseline level to demonstrate the relationship between reward sensitivity slopes. Error bars indicate standard error of the mean difference observed between each reward level and the 0p baseline. (**B**) Pupil reward sensitivity across the groups, calculated as the difference in pupil response between 50p and 0p rewards on offer. Box and whisker plots indicate median (line within box), mean (+), interquartile range (box outline) and maximum and minimum values (whiskers).

Pupillary change in relation to rewards offered was examined in each group separately using a repeated-measures ANOVA. The average pupil proportional change for each reward offered was measured over the 1400–2400 ms epoch of interest. This revealed a significant effect of reward in both healthy controls [*F*(1.8,73.3) = 16.9, *P* < 0.001] and iRBD patients [*F*(1.8,73.7) = 6.8, *P* = 0.003], but not in Parkinson’s disease patients [*F*(1.5,56.6) = 2.5, *P* = 0.10] (Fig. 3A). Including all the groups and after controlling for age, gender and average baseline pupil size, a significant group by reward level interaction was found [*F*(3.6,209.9) = 4.5, *P* = 0.002]. To deconstruct this finding, pRS (the difference or slope between pupil response at 0p and 50p levels over the specified time period of 1400–2400 ms) was assessed. This demonstrated a stepwise decline in pRS, highest in healthy controls and reducing in RBD and Parkinson’s disease patients (Fig. 3B).

In Parkinson’s disease patients, the 95% CI of the mean pRS crosses zero (mean 0.12, 95% CI −0.07–0.31), indicating that pupil modulation was no more responsive to 50p than to 0p. Pairwise comparisons between the groups (adjusting for age, gender and average pupil size) showed that pRS was significantly lower in RBD patients than controls (*P* = 0.04) and significantly lower in Parkinson’s patients than controls (*P* = 0.002) but not significantly different between iRBD and Parkinson’s patients.

### Effect of dopaminergic deficit on reward sensitivity in RBD patients

Eighteen out of 41 iRBD patients had abnormal DaT SPECT/CT imaging as classified by the blinded radiologist assessment. Idiopathic RBD patients with imaging classed as abnormal had significantly lower mean dopaminergic specific uptake ratio (SUR) in the putamen than those with normal imaging (group means: abnormal, SUR = 1.81 versus normal, SUR = 2.35, *t*-test, *P* < 0.001; Supplementary Fig. 1).

**Figure 4:**
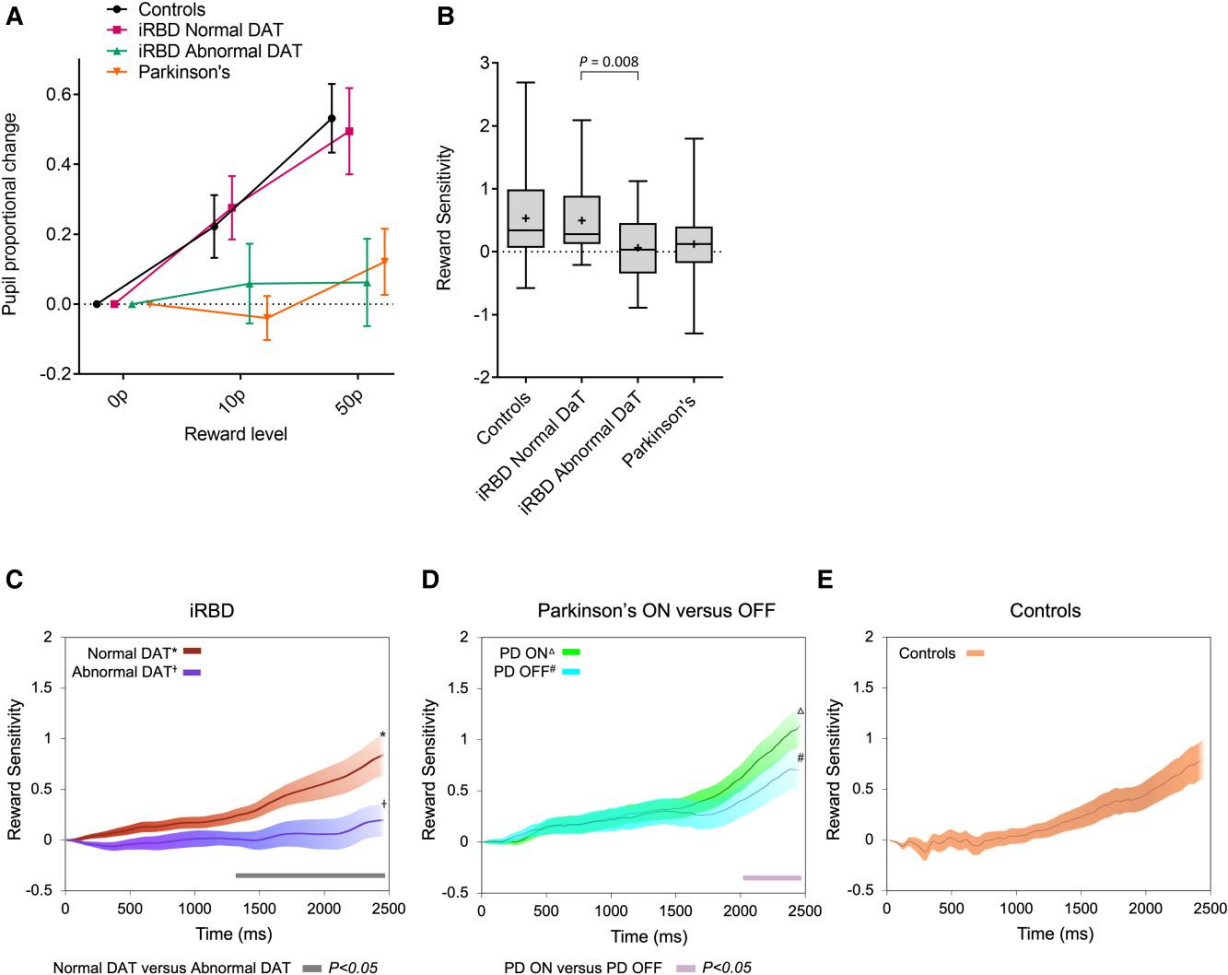
**Pupillary response to reward in controls, Parkinson’s disease patients and iRBD patients split according to abnormal and normal DaT SPECT/CT imaging.** (**A**) Proportional pupil changes at each reward level across healthy controls, Parkinson’s disease patients and RBD patients divided into DaT SPECT/CT outcome, with each group normalized to the 0p level. Error bars indicate standard error of the mean difference observed between each reward level and the 0p baseline. (**B**) Pupil reward sensitivity (pRS) across the groups including RBD subgroups, calculated as the difference in pupil response to 50p and 0p. Box and whisker plots indicate median (line within box), mean (plus sign), interquartile range (box outline) and maximum and minimum values (whiskers). (**C**–**E**) Mean pRS (pupil change to 50p reward minus response to 0p) plotted over time in RBD patients (**C**), Parkinson’s disease patients (**D**) and controls (**E**). (**C**) In RBD patients, a significant difference in pRS between those with normal (red, asterisk) and abnormal (purple, plus sign) dopaminergic imaging occurred from ∼1300 ms to the end of the trial, indicated by the grey bar (*P* < 0.05). (**D**) In Parkinson’s patients, there was a significant reduction in pRS when off dopaminergic medication (blue, number sign) versus on (green, triangle). Parts **D** and **E** adapted from previously published data (Muhammed *et al.*).^1^ Shaded areas indicate standard error of the mean.

The effect of dopaminergic depletion in iRBD on pupillary changes in relation to rewards offered was assessed. A repeated-measures ANOVA including both iRBD subgroups demonstrated a significant reward by DaT SPECT/CT group interaction [*F*(1.9,69) = 4.4, *P* = 0.017], indicating that the differential response to reward level is related to DaT SPECT/CT abnormality (Fig. 4A, green versus purple lines). Examination of each group of iRBD patients separately revealed a significant effect of reward level in the normal DaT SPECT/CT group [*F*(1.8,39.2) = 10.0, *P* < 0.001] (Fig. 4A, steep purple slope) but not in the iRBD patients with abnormal DaT SPECT/CT imaging [*F*(2.0,33.6) = 0.2, *P* = 0.83] (Fig. 4A, shallow green slope).

Pairwise comparisons of pRS revealed iRBD patients with abnormal DaT SPECT/CT imaging had significantly lower pRS than those with normal imaging (*P* = 0.008) (Fig. 4B). Idiopathic RBD patients with abnormal imaging were comparable to Parkinson’s patients, showing no significant difference in pupil response between 0p and 50p. Idiopathic RBD patients with normal imaging were indistinguishable from healthy controls.

pRS over time was also analysed for RBD patients with normal or abnormal DaT SPECT/CT imaging (Fig. 4C). Using permutation testing, mean pRS in the iRBD subgroups confirmed a significant difference from ∼1300 ms to the end of the trial (duration denoted by the grey bar). Previously published data showing pRS over time in Parkinson’s patients on and off dopaminergic medication (Fig. 4D) and in controls (Fig. 4E) is displayed for comparison. Parkinson’s disease patients showed increased pRS in the on-medication state compared to the off state from 2000 ms to the end of the trial.^1^

**Figure 5:**
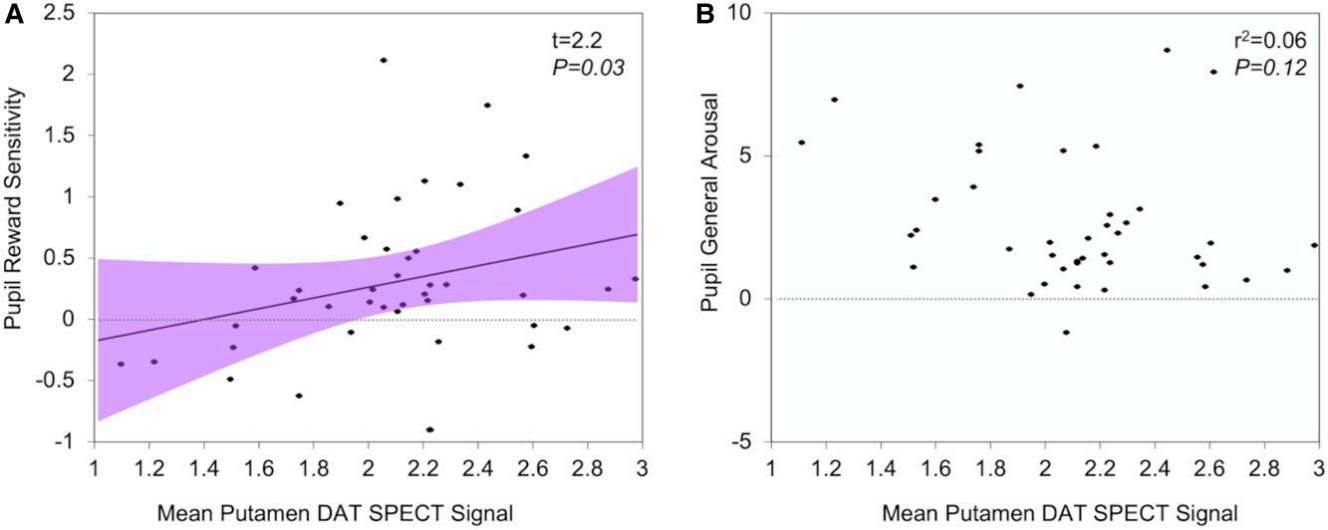
**Association between average putamen DaT SPECT/CT signal, pRS and average pupillary arousal level.** (**A**) Association between pRS and mean putamen DaT imaging. Linear mixed-effects modelling was used to encompass reward sensitivity scores while including 0p, 10p and 50p data. A significant positive association between pRS and mean putamen DaT SPECT/CT signal was demonstrated (*t* = 2.2, *P* = 0.03). Purple shaded area indicates 95% CI of the best fit line. (**B**) General pupil arousal was measured as the average change in pupil response across all reward levels to the cue over the 1400–2400 ms period of interest. This was correlated against an individual’s mean putamen DaT SPECT/CT signal and no significant effect was found.

The effect of dopamine depletion on pRS in iRBD patients was also assessed using the mean DaT SPECT/CT SURs in the putamen as a continuous measure. Repeated-measures ANOVA with pupil change for each reward level and putamen uptake ratio in iRBD patients was performed. A significant reward level-by-mean putamen signal interaction was found [*F*(1.9,69.7) = 4.1, *P* = 0.021]. Since reward sensitivity is measured as the pupillary response change in 50p versus 0p reward over the 1400–2400 ms period of interest, the effect of the middle 10p reward level is not included in this metric. Therefore, to encompass all the data, a linear mixed-effects analysis was performed. A significant positive association between pRS and mean putamen DaT SPECT/CT signal was found (*t* = 2.2, *P* = 0.03; Fig. 5A), while no correlation between pupillary arousal (average pupillary response to all reward levels) and mean putamen DaT SPECT/CT signal was demonstrated (*r* = −0.246, *P* = 0.12; Fig. 5B).

### Relationship between apathy, depression and reward sensitivity

In patients with Parkinson’s disease, we have previously demonstrated an association between pRS and apathy severity (measured using LARS score).^1^ This association was not observed in iRBD patients here. There was no significant correlation between LARS scores and pRS (*r* = 0.02, *P* = 0.88) in iRBD and no difference in pRS between patients who met the LARS threshold (total score ≥ −21) for clinical apathy and those who did not (*P* = 0.64). Differences in pRS were also not explained by levels of depression, with no correlation observed between Beck Depression Inventory scores and reward sensitivity among iRBD patients (*r* = −0.02, *P* = 0.91).

### Baseline pupil and oculomotor parameters

Average baseline pupil size (ANOVA, *P* = 0.61) and average pupil proportional change across all three reward levels (general pupillary arousal) (ANOVA, *P* = 0.10) did not differ significantly between the three groups of participants, suggesting that resting autonomic tone at the pupillary muscles and general pupil responsiveness were no different between controls, iRBD patients and Parkinson’s disease patients.

No difference in baseline pupil size (*β* = −0.12, *P* = 0.47) or average general pupil arousal was found between iRBD patients with normal versus abnormal imaging (*β* = 0.20, *P* = 0.17). This implies that the group difference in pRS is not due to generally more responsive pupils in patients with normal imaging.

There were no significant differences in average RT (*β* = 0.07, *P* = 0.67) nor average peak saccadic velocity (*β* = 0.13, *P* = 0.43) between the iRBD patients with abnormal or normal DaT imaging, indicating that their task performance was comparable.

## Discussion

To our knowledge, this study is the first to demonstrate that pupillary responses to reward are impaired in patients with iRBD and further that this is associated with dopamine depletion in the striatum. Idiopathic RBD patients as a group showed similar levels of pRS to Parkinson’s disease patients, which were reduced compared with healthy controls (Fig. 3B). This reduction in iRBD patients was accounted for by those with abnormal dopaminergic imaging; iRBD patients with normal imaging were indistinguishable from controls in pRS (Fig. 4B). Importantly, there were no significant differences in general pupillary arousal or task performance between iRBD patients with normal versus abnormal dopaminergic imaging, suggesting a specific effect on reward processing rather than dysfunction in autonomic or arousal pathways.

### Dopamine depletion and reward sensitivity in prodromal disease

Previous work has demonstrated that exogenous dopamine can improve reward sensitivity in Parkinson’s patients, implying that dopaminergic deficits may be involved in the mechanisms underlying insensitivity to reward (Fig. 4D).^1^ This study builds on these data by demonstrating a direct link between brain dopamine availability and reward responsiveness in patients with iRBD. Dopaminergic degeneration is usually much less extensive in iRBD patients than in those with established Parkinson’s disease and occurs many years before motor parkinsonism develops,^10,20^ suggesting that pupillary reward responses may be sensitive to even modest reductions in dopamine availability, early in the prodromal disease stage. Examining this association during prodromal disease also removes the potential confounding effect of dopaminergic medications when studying Parkinson’s patients. Since individuals with iRBD have never received such treatments, we are able to investigate the effects of dopaminergic neurodegeneration in the natural state.

While dopamine depletion in the putamen was measured in this study, we do not suggest that this region itself is the key mediator in reward appraisal. The mesolimbic dopaminergic pathway, projecting from the ventral tegmental area to the ventral striatum, has a more important mechanistic role than the nigro-striatal pathway in the evaluation and processing of reward.^21,22^ Although the mesolimbic pathway is known to be affected by Parkinsonian neurodegeneration,^23^ measurements of dopaminergic integrity in the putamen are more readily accessible using DaT SPECT and may be more sensitive measures of prodromal dopamine deficit since this region is proportionally affected more in early disease.^24,25^ When considering its relationship with reward sensitivity, our measurement of nigro-striatal integrity may therefore be a surrogate marker of dysfunction in the mesolimbic pathway. Degeneration in non-dopaminergic pathways, including the noradrenergic system, may also be involved in changes to pupil responses.^26^ However, the finding that putamen DaT SPECT/CT signal did not correlate with average pupil responsiveness suggests that the observed relationship between reward sensitivity and dopaminergic signal is unlikely to be explained by an effect of neurodegeneration on levels of arousal or autonomic function.

It should be noted that the comparison between iRBD patients as a prodromal group and patients with Parkinson’s is in some respects an oversimplification. It is well established from longitudinal studies that iRBD patients phenoconvert in approximately equal proportions to Parkinson’s disease and DLB, with a much smaller proportion converting to multiple system atrophy.^7^ Furthermore, evidence from Parkinson’s populations increasingly highlights iRBD as a marker of a more diffuse and rapidly progressive disease subtype, with more extensive nigro-striatal dopaminergic deficit.^20,27^ Therefore, while our iRBD patients are presumed to be at an earlier stage of nigro-striatal degeneration than our Parkinson’s patients, as a group they may have a more aggressive and/or diffuse form of synucleinopathy. In support of this, we have previously demonstrated that non-motor features are at least as severe in iRBD as in established Parkinson’s and that certain neuropsychiatric symptoms are even more prominent.^11^

The concept of iRBD as a more malignant prodromal synucleinopathy may be relevant to our observation that iRBD patients with a dopaminergic deficit show similar levels of reward insensitivity to patients with established Parkinson’s disease (Fig. 4B), rather than an intermediate level that might be expected during the prodromal phase. Although iRBD patients are presumed to be at an earlier stage of dopaminergic decline, it is possible that other neurotransmitter systems involved in response to reward may already be equally (or more) compromised. Another possibility is that there is a floor effect during the prodromal phase, with pRS already maximally reduced by the time a nigro-striatal dopaminergic deficit becomes apparent with DaT SPECT/CT imaging. Last, the patients with Parkinson’s disease in this study were treated with dopaminergic therapy. Even in the off state, following an overnight hold of medication, residual dopamine may still affect pupil responses and contribute to the similar level of reward sensitivity seen when off compared to iRBD with abnormal DaT SPECT/CT.

### Reward insensitivity as a potential prodromal risk marker

Our findings raise the possibility that pRS metrics might have value as a risk stratification marker in iRBD patients. Numerous studies have demonstrated that up to half of patients with iRBD have a measurable dopaminergic deficit in the basal ganglia, and there is clear evidence that these patients have a higher short-term risk of converting to an overt synucleinopathy.^7,10^ However, previous research has been unable to identify clinical markers corresponding to this deficit during prodromal disease.^9,12,28^ Biometric technology has emerged as a potential solution to this problem and techniques using computer and smartphone-based assessments are increasingly being introduced to measure motor and cognitive decline.^29,30^ Quantifying an individual’s sensitivity to reward in this way using eye-tracking linked to physiological responses is now more feasible and, despite some technical challenges, might prove to be a practical way of gaining insight into an individual’s dopaminergic status. While these methodologies will not supplant neuroimaging as a definitive test, in combination with other tests of Parkinsonian features, measuring pRS could form an important part of a multimodal risk stratification model. Several questions remain to be addressed before translation to the clinic can occur. Longitudinal studies are needed to establish whether iRBD patients with reward insensitivity are indeed at greater risk of near-term pheno-conversion, and whether changes in pupil metrics over time can be used on an individual basis to measure progressive dysfunction in reward processing. Replication of our findings in distinct populations will also be important. Finally, wider implementation will require adaptation of the ocular task away from the specialist laboratory setting and towards in-clinic, or even remote, device-based measurement. Smartphones, with their increasingly sophisticated front-facing cameras, have great potential to facilitate this, and the development of applications that can index pupil responses to on-screen tasks might be one way in which our findings could be more widely translated.

### Reward insensitivity and clinical apathy

In this group of iRBD cases, we found no relationship between pRS and measures of apathy or depression. The finding with respect to apathy is in contrast to the inverse relationship between apathy severity and pRS that has been observed in Parkinson’s patients in previous studies.^1,2^ These findings contribute to an emerging picture of reward-based decision making in Parkinson’s disease in which apathy and dopaminergic mechanisms are partially dissociable.^5,31–34^ In a recent study comparing apathetic and non-apathetic Parkinson’s disease patients, both on and off dopaminergic medication, Le Heron and colleagues^32^ demonstrated that although apathy and dopamine depletion were both associated with a reduction in willingness to exert effort for reward, the mechanisms were different. Apathy resulted in the increased rejection of low value rewards, while dopamine increased responses to high rewards requiring high effort in both apathetic and non-apathetic patients. In a separate study, dopamine was again shown to increase effort-based decisions in Parkinson’s disease patients despite the absence of clinical apathy.^5^ As with our findings, this implies that dopamine-dependent mechanisms involved in motivational deficits may be subclinical and not sufficient to cause overt clinical apathy. Taken together, these results suggest that while exogenous dopamine may have a generalized invigorating effect on patients, whatever their motivational state, distinct mechanisms may underlie clinical apathy, of which reward sensitivity related to dopaminergic modulation is one contributory factor. Another mechanism may involve serotonergic pathways. Depletion of serotonin has been correlated with clinical apathy in early Parkinson’s disease using specific neuroimaging techniques.^35^ We have previously demonstrated a relationship between apathy and serotonergic signal in the dorsal raphe nucleus in iRBD.^36^ It seems likely that the clinical syndrome of apathy in Parkinsonian disorders involves disruptions in several distinct neurotransmitter systems as well as the interactions between them. The relative contributions from different networks may vary according to disease stage as neurodegeneration progresses. The findings presented here suggest that a dopamine-dependent component may be detectable early in the development of Parkinson’s disease while still clinically silent.

### Limitations of our study

Some limitations of our study should be noted. Due to the high male to female ratio observed in iRBD cohorts, our iRBD group was not matched for sex with the previously published control and Parkinson’s groups. While we included sex as a covariate in our analyses to control for this, we cannot fully exclude sex differences contributing to our findings. Only iRBD patients underwent SPECT/CT imaging, meaning that imaging abnormality for dichotomized analyses was defined by expert opinion rather than quantitative comparison with control imaging. However, as subtle early abnormalities may relate to the signal pattern as much as absolute SUR values, we consider this to be an equally valid classification method. Furthermore, the results of our dichotomized analyses were supported by findings using the objectively quantified putamen SUR (Fig. 5). As Parkinson’s patients did not undergo imaging, we cannot be sure that they had more extensive dopaminergic deficits than our RBD patients, although this has been clearly demonstrated in other studies comparing DaT SPECT in iRBD and Parkinson’s.^20^ Neither control nor Parkinson’s patients underwent polysomnography to determine the presence or absence of RBD. This is unlikely to have significantly affected the control versus iRBD comparison, as iRBD is relatively rare in the general population.^37^ As noted before, this does to some extent limit the comparison between iRBD and Parkinson’s patients, as Parkinson’s patients without RBD may have a more benign disease phenotype than those with concomitant RBD. However, the main purpose of this comparison for our study concerns the degree of dopaminergic deficit, which is expected to be lower in Parkinson’s patients than iRBD patients, whether or not concomitant RBD is present.^20^ Finally, we did not include patients with DLB, which is a phenoconversion outcome as likely as Parkinson’s disease for iRBD patients.^7^ Studying pupil responses in a behavioural task such as this would be challenging and difficult to interpret in patients with dementia, and this was beyond the scope of our study. However, as DaT SPECT/CT findings are similar in Parkinson’s and DLB,^15,38^ and both would be expected to show more advanced dopaminergic deficits than iRBD, we believe the comparison between iRBD and Parkinson’s patients alone remains valid when considering dopaminergic phenomena.

## Conclusion

Our data demonstrate that reward sensitivity, as indexed by pupil responses, is reduced in iRBD patients and correlates with striatal dopamine availability. As well as providing evidence for the role of dopaminergic transmission in reward evaluation, these findings imply that impaired pupillary response to reward may be a marker of early striatal neurodegeneration. Unlike in established Parkinson’s disease, reward insensitivity does not relate to clinical apathy in iRBD patients, suggesting that the aetiology of apathy may vary with disease stage and may not be accounted for by disruption in reward evaluation pathways alone.

## Supplementary Material

awac430_Supplementary_Data
