# Supplementary material for: Reward insensitivity is associated with dopaminergic deficit in rapid eye movement sleep behaviour disorder
Source: Brain. 2022 Nov 17;146(6):2502–11. doi: 10.1093/brain/awac430 (PMC10232265; doi:10.1093/brain/awac430)
Supplement: awac430_Supplementary_Data [file awac430_supplementary_data.pdf]

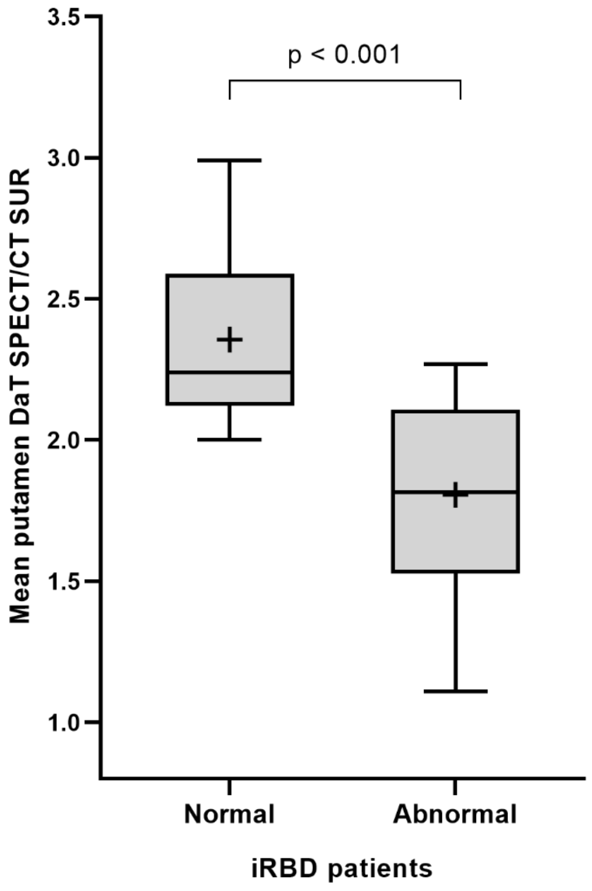

Supplementary figure 1. Mean DaT SPECT/CT SUR from the putamen in iRBD patients according to classification of imaging by the clinical radiologist blinded assessment. iRBD patients with DaT SPECT/CT imaging classed as abnormal had significantly lower SUR than those with normal imaging. Box and whisker plots indicate median (line within box), mean (+), interquartile range (box outline), maximum and minimum values (whiskers).

Abbreviations: DaT SPECT/CT, dopamine transporter single photon emission computed tomography with CT attenuation correction; SUR, specific uptake ratio; iRBD, idiopathic rapid eye movement sleep behaviour disorder.
